# Editorial: Enhancing kidney transplant outcomes through machine learning innovations

**DOI:** 10.3389/frai.2025.1760127

**Published:** 2025-12-19

**Authors:** Antonio Sarasa-Cabezuelo, Krishna Kumar Sharma, Amado Andrés Belmonte, Ana M. Gonzalez De Miguel, Ulises Roman-Concha

**Affiliations:** 1 Departamento de Sistemas Informáticos y Computación, Complutense University of Madrid, Madrid, Spain; 2 Department of Computer Science, University of Kota, Kota, India; 3 Departamento de Medicina, Complutense University of Madrid, Madrid, Spain; 4 Departamento Ingeniería del Software e Inteligencia Artificial, Complutense University of Madrid, Madrid, Spain; 5 Departamento de Ciencias de la Computación, National University of San Marcos, Lima, Peru

**Keywords:** artificial intelligence, graft survival, kidney transplantation, machine learning, organ allocation, predictive modeling

Chronic kidney disease represents one of the most significant health challenges of our time, affecting more than 850 million people worldwide. Kidney transplantation remains the optimal renal replacement therapy, offering better clinical outcomes and quality of life compared to dialysis. However, the inherent complexity of transplant decision-making, from organ allocation to post-transplant follow-up, demands increasingly sophisticated predictive tools. In this context, machine learning is emerging as a transformative technology capable of revolutionizing how we approach kidney transplant outcomes.

This Research Topic brings together six original contributions demonstrating how innovations in machine learning can significantly improve different aspects of the transplantation process, from predicting graft failure to optimizing organ allocation.

## Graft failure prediction: a dynamic approach

The ability to predict kidney graft failure at different post-transplant phases is critical for timely intervention. Salybekov et al., in their work “*Phase-Specific Kidney Graft Failure Prediction with Machine Learning Model*,” developed machine learning models specific to five post-transplant time intervals. Their results reveal that predictive accuracy varies according to the analyzed period, reaching its maximum performance in the 9- to 15-month window (ROC AUC = 0.92), suggesting that graft failure risk patterns evolve dynamically. This contribution underscores the importance of adapting surveillance strategies to the different phases of post-transplant follow-up.

Complementing this temporal perspective, He et al. present, in “*A Machine Learning-based Nomogram for Predicting Graft Survival in Allograft Kidney Transplant Recipients: A 20-year Follow-up Study*,” a nomogram based on LASSO regression techniques, random survival forest, and Cox regression analysis. With a concordance index of 0.827, the model identifies six key predictive factors, including recipient cardiovascular disease, delayed graft function, serum phosphorus, donor age, donor creatinine, and donation after cardiac death. This study, with a two-decade follow-up, provides a clinically applicable tool for individualized assessment of long-term prognosis.

## Optimizing organ allocation

One of the most pressing challenges in transplantation is the underutilization of kidneys from deceased donors. Berry et al., in “*Predicting Offer Burden to Optimize Batch Sizes in Simultaneously Expiring Kidney Offers*,” address this problem using machine learning-based survival models to dynamically predict the number of offers a kidney will require before being accepted. Their Random Survival Forest model achieved a time-dependent C-index of 0.882, demonstrating the potential to reduce placement times from 17.37 h to just 1.59 h, an improvement that could have substantial implications for organ viability and transplant outcomes.

Similarly, Li et al. present simplified but effective models for predicting the risk of kidney non-use in “*Improving Deceased Donor Kidney Utilization: Predicting Risk of Nonuse with Interpretable Models*.” Their approach, which combines the Kidney Donor Risk Index with a small set of variables selected using machine learning, demonstrates that interpretability does not necessarily compromise predictive performance. Furthermore, their analysis incorporates factors at the Organ Procurement Organization level, revealing significant variations in kidney disposal practices among different organizations.

## Perioperative complications and follow-up biomarkers

Sun et al. expand the scope of machine learning in transplantation with their work “*Machine Learning-based Predictive Model for the Perioperative Co-occurrence of T-cell-mediated Rejection and Pneumonia in Liver Transplantation*.” Although focused on liver transplantation, this study illustrates the cross-cutting applicability of ML for predicting complex post-transplant complications. Their support vector machine model achieved an AUC of 0.881, and the use of the SHAP method to visualize the contributions of variables represents an important step toward more transparent and interpretable models.

Finally, Batko et al. in “*Risk Prediction of Kidney Function in Long-term Kidney Transplant Recipients*” provide a complementary perspective by examining the role of emerging biomarkers such as elabela, apelin, FGF-23, and α-Klotho in long-term kidney transplant recipients. Their longitudinal study of 102 patients who maintained graft function for at least 24 months reveals significant alterations in these markers compared with healthy controls, establishing a basis for future research on biomarker-based risk stratification.

## Future perspectives

The contributions gathered in this Research Topic collectively demonstrate that machine learning is maturing as a clinical tool in transplantation. However, significant challenges remain: the need for multicenter external validation, effective integration into existing clinical workflows, and ethical considerations related to data privacy and algorithmic fairness.

Interpretability emerges as a recurring theme in several presented works, reflecting the growing awareness that black box models have limitations in clinical contexts where decisions must be explainable and justifiable. Methods such as SHAP and nomogram-based approaches represent significant steps toward more transparent decision support systems.

This Research Topic aims to inspire further research at the intersection of machine learning and transplant medicine, fostering interdisciplinary collaborations that accelerate the translation of these technological innovations into tangible improvements for patients awaiting or who have received a kidney transplant.

